# Identification of an Autoantibody Against ErbB-3-Binding Protein-1 in the Sera of Patients With Chronic Hepatitis B Virus Infection

**DOI:** 10.3389/fimmu.2021.640335

**Published:** 2021-05-25

**Authors:** Li Jiang, Wei Niu, Qian Zheng, Gang Meng, Xiaoling Chen, Mengjun Zhang, Guohong Deng, Qing Mao, Li Wang

**Affiliations:** ^1^ Department of Infectious Diseases, Southwest Hospital, Army Medical University (Third Military Medical University), Chongqing, China; ^2^ Department of Immunology & Institute of Immunology, Army Medical University (Third Military Medical University), Chongqing, China; ^3^ Function Center, North Sichuan Medical College, Nanchong, China; ^4^ Department of Pathology, Southwest Hospital, Army Medical University (Third Military Medical University), Chongqing, China; ^5^ Department of Pharmaceutical Analysis and Analytical Chemistry, College of Pharmacy, Army Medical University (Third Military Medical University), Chongqing, China

**Keywords:** liver damage, chronic hepatitis B, autoreactive T-cell response, autoantibody, autoantigen, ErbB-3-binding protein-1

## Abstract

**Background:**

Studies have shown that autoimmune response contributes to chronic hepatitis B (CHB) development.

**Aim:**

This study aimed to identify autoantibodies in the sera of patients with CHB and to investigate the association of autoimmune response with disease severity in CHB.

**Methods:**

Proteins from human liver carcinoma cell line HepG2 were separated by two-dimensional electrophoresis. The candidate autoantigens were recognized by serum autoantibodies from Chinese CHB patients. Immunohistochemical staining was performed to determine the hepatic expression of the autoantigen in CHB patients with different inflammatory grades. Enzyme-linked immunosorbent assay (ELISA) was conducted to measure the prevalence and the levels of serum autoantibody in CHB patients with different disease severity. Flow cytometry analysis was carried out to assess the autoreactive T cell response in the peripheral circulation of CHB patients.

**Results:**

ErbB-3-binding protein-1 (EBP-1) was identified as an autoantigen of serum autoantibodies in CBP patients. EBP-1 protein expression was upregulated in the liver of CHB patients with high-grade hepatic inflammation. The prevalence and levels of serum anti-EBP1 IgG were significantly increased in CHB patients with severe diseases compared with those with mild or moderate diseases, but none was detectable in the healthy controls. EBP-1 peptides induced proinflammatory cytokine expression in CD4^+^ T cells from CHB patients.

**Conclusion:**

Our results demonstrate the presence of an autoantibody against EBP-1 in the sera as well as EBP-1-reactive T cells in the peripheral blood of CHB patient. EBP-1-induced autoimmune response is positively associated with the disease severity, suggesting that EBP-1-induced autoimmune response possibly contributes to progressive liver failure.

## Introduction

Hepatitis B virus (HBV) infection is a major global public health problem that affects more than 240 million individuals worldwide, leading to chronic hepatitis B (CHB), cirrhosis, and hepatocellular carcinoma (1, 2). CHB is characterized by liver inflammation and deterioration of liver function reflected by biochemical abnormalities, such as increased serum levels of alanine aminotransferase (ALT) and total bilirubin (TB) and decreased prothrombin time activity (PTA) (3). The specific cellular immune responses to HBV contribute to both viral clearance and liver injury in CHB (4, 5); however, the immune mechanisms of liver damage in CHB are not completely understood.

Autoimmunity has been implicated in CHB. The liver histological features of CHB, such as portal and periportal lymphocyte infiltration and piecemeal necrosis of periportal hepatocytes (6), are very similar to those of autoimmune hepatitis (AIH) mediated by liver-targeting autoantibodies and autoreactive T cells (7). Indeed, autoantibodies are common in CHB. Liver antigen-specific autoantibodies have been shown to contribute to periportal liver damage in CHB (8). Several non-organ-specific autoantibodies, such as nuclear antibodies, smooth muscle antibodies, and liver/kidney microsomal antibody, have been detected in CHB patients. However, the associations of the autoantibodies with liver damage remain unclear (9, 10). Autoantibody production by B cells requires help from autoantigen-activated CD4^+^ T cells. Studies have demonstrated that the autoantigens targeted by autoantibodies can also be recognized by autoreactive T cells in human (11, 12). Studies on mouse hepatitis models have shown that the recruitment of non-HBV-specific bystander T cells contribute to the liver damage in CHB through creating a proinflammatory microenvironment (13–16). Thus, we hypothesized that autoantigen/autoantibody-induced autoimmune response may contribute to liver inflammation in CHB and that the bystander T cells might represent a population of liver antigen-autoreactive T cells in CHB.

To test our hypothesis, we sought to identify liver autoantigens and serum autoantibodies in a cohort of Chinese CHB patients and explored the association of autoimmune response with the severity of disease. We also investigated autoreactive T-cell responses in CHB patients. Our study may provide new insights into the pathogenesis of CHB and novel immunotherapeutic target for CHB treatment.

## Materials and Methods

### Subjects

This study was approved by the Ethics Committee of Southwest Hospital (Chongqing, China). All procedures were in compliance with the Good Clinical Practice Guidelines and the 1975 Declaration of Helsinki. A total of 150 patients with CHB and 59 age- and gender-matched healthy controls (HCs) were recruited from Southwest Hospital (**Table 1**). All participants provided written informed consent before study initiation. The diagnosis was based on EASL 2017 Clinical Practice Guidelines on the management of hepatitis B virus infection (17) and the Guidelines For Diagnosis And Treatment Of Liver Failure (2018 version). Subjects coinfected with human immunodeficiency virus or hepatitis C virus, or hepatitis D virus, diagnosed with autoimmune or alcoholic hepatitis, or currently treated with immunosuppressive or antiviral therapy were excluded. Other serum samples were collected from 24 untreated patients with chronic hepatitis C (CHC), 23 with autoimmune hepatitis (AIH), all fulfilling the diagnostic criteria. Serum samples were obtained from all subjects and stored at −80°C until use. Fresh peripheral blood mononuclear cells (PBMCs) were obtained from 62 CHB patients and 35 HCs to measure antigen-specific T cell responses.

**Table 1:** Clinical characteristics of patients and HCs.

	Healthy controls (HC)	chronic HBV infection(CHI)^*^	chronic hepatitis B(CHB)^**^	HBV-associated acute-on-chronic liver failure(ACLF)^***^
Number of subjects	59	48	72	30
Gender (male/female)	42:17	27:21	55:17	27:3
Age (years)	36 (19-60)	35 (20-67)	40 (18-73)	41.8 (23-67)
alanine aminotransferase, units/L	ND	26 (12-42)	525.56 (43-3016)	743.28 (53-4113)
HBV DNA, IU/mL	ND	3.26×10^6^ (60-3.02×10^7^)	4.39 ×10^6^ (60-5.58×10^7^)	6.48×10^6^ (60-9.26×10^7^)
Total bilirubin (TB) μmol/L	ND	10.2 (4.6-17.0)	90.83(11.3-509)	385.52 (175.2-881.2)
prothrombin time (PT) second	ND	11.6 (8.7-14.0)	13.41 (9.2-19.6)	27.07 (19.0-56.4)
International Normalized Ratio(INR)^****^	ND	0.9 (0.7-1.1)	1.1(0.7-1.6)	2.2(1.5-4.5)
HBeAg positive Count(%)	0	30 (62.5%)	31 (43.06%)	11 (36.67%)

^*^CHI includes HeAg-positive or HeAg-negative chronic HBV infection. Their liver function was normal, but HBV DNA was detectable.

^**^CHB includes HeAg-positive or HeAg-negative chronic hepatitis B. It was composed of mild, moderate and severe hepatitis.

^***^ACLF is diagnosed when both INR≥1.5 and TB≥171μmol/L.

^****^International normalized ratio (INR)=(PT_test_/PT_normal_)^ISI^, ISI, International Sensitivity Index.

Data are shown as median and range. ND, not determined.

A total of 36 CHB patients underwent liver biopsy for histopathology and immunohistochemical (IHC) assays. The normal liver tissue samples were obtained by surgical resection from the patients diagnosed as intrahepatic bile duct stones or hemangioma. Hepatic inflammation was graded using the modified histological activity index (HAI) (10).

### Cell Culture

Human liver hepatoma cell line HepG2 (ATCC) was maintained in Dulbecco’s Modified Eagle’s Medium (DMEM) supplemented with 2 mM L-glutamine, sodium pyruvate, nonessential amino acids, 100 U/mL penicillin, 100 mg/mL streptomycin, and 10% fetal bovine serum (FBS) at 37°C in a humidified atmosphere of 5% CO_2_.

### PBMC Preparation

Peripheral venous blood (6 mL) from CHB patients or HCs were collected into a heparin anticoagulation tube and mixed with an equal volume of phosphate-buffered saline (PBS) at room temperature. PBMCs were isolated by density gradient centrifugation using LymphoprepTM (Axis-Shield PoC AS, Norway). Briefly, the diluted blood sample was added into 6 mL lymphocyte separation solution Ficoll, followed by centrifugation at 2,000 rpm for 30 min at 18–20°C. After removing red blood cells, granulocytes, stratified liquid, and plasma, PBMCs were mixed with at least 3 × volumes of PBS, followed by 2 times of centrifugations at 2,000 rpm for 10 min at 18–20°C. After removing the supernatant, PBMCs were resuspended in 1 mL RPMI-1640 medium containing 10% FBS.

### Two-Dimensional Electrophoresis (2-DE)-Based Serologic Proteomic Analysis

To identify liver proteins recognized by serum autoantibodies from CHB patients, a serologic proteomic analysis was performed as previously described (18). The reagents were purchased from Amersham Pharmacia Biotech (Uppsala, Sweden). Briefly, HepG-2 cell lysates were separated by 12.5% SDS-PAGE gels, and the proteins were transferred to a polyvinylidene difluoride (PVDF) membrane or visualized using Coomassie blue. The PVDF membrane was incubated with a mixture of diluted serum samples (1:200) from 20 CHB patients or 15 HCs, followed by incubation with horseradish peroxidase (HRP)-conjugated goat anti-human IgG (1:10,000; Beyotime, Shanghai, China). The results were visualized using an enhanced chemiluminescence reagent (Roche, Basel, Switzerland) and compared with Coomassie-stained blots to determine the immunoreactive proteins. Images were acquired using a GS-800 Calibrated Densitometer (Bio-Rad, Hercules, CA, USA). Spots of interest were digested with trypsin for 16 h at 37°C. The resulting peptides were concentrated and desalted using a ZipTip μ-C18 column (Millipore, Bedford, MA, USA) and analyzed using a matrix-assisted laser desorption ionization time-of-flight mass spectrometer (Burker Company, Germany). The spectra were analysed using the Mascot Search engine and the SwissProt database.

### Western Blot Analysis

Recombinant human ErbB-3-binding protein-1 (rhEBP-1) (purity > 95%; Novus Biologicals, Littleton, CO, USA) was detected by the diluted serum samples (1:100) from CHB patients or HCs or by a mouse polyclonal anti-human ErbB-3-binding protein-1 (EBP-1) IgG (1:500; Novus Biologicals). The membranes were incubated with HRP-conjugated goat anti-human (1:10,000; Beyotime) or anti-mouse IgG (1:5000; Beyotime). The protein bands were visualized using a BeyoECL Plus system (Beyotime).

### IHC Staining of EBP-1 in Liver Tissue Samples

The formalin-fixed, paraffin-embedded tissue sections were deparaffined and rehydrated. After quenching endogenous peroxidase activity and antigen retrieval, the slides were incubated with blocking buffer (Beyotime) and incubated with rabbit anti-EBP-1 polyclonal antibody (1:200; Novus Biologicals) or normal rabbit IgG (1:200) overnight at 4°C, followed by incubation with goat anti-rabbit IgG-HRP (1:100; Beyotime). Bound peroxidase was visualized with 3, 3’-diaminobenzidine (Beyotime). The intensity of EBP-1 was scored as follows: 0 = no color, 1 = light yellow, 2 = light brown, and 3 = brown. Nuclei were counterstained with hematoxylin. The slides were observed using a light microscope.

### ELISA

The recombinant human EBP-1 protein (Novus Biologicals) was served as the detection antigen, the optimal working concentrations of coating antigen and the dilution of serum samples were determined by chessboard titration, and the reaction condition was optimized, based on which an indirect ELISA method was developed and verified. Briefly, each well of a multi-titre ELISA plate (Dynatech, Alexandria, VA, USA) was coated with 100μl 2μg/ml rhEBP-1 in 50 mmol/L sodium carbonate (pH 9.6). Non-specific reactivity was blocked with 10% fetal calf serum in PBS. The serum samples (1:200) were incubated for 1 h, and bound antibodies were detected using HRP-conjugated goat anti-human IgG (Beyotime). The optical density at 450 nm was measured using a microplate reader (Bio-Rad). The antibody titre was expressed as arbitrary binding units calculated as [ODsample/(meanOD_HC_ + 2SD of OD_HC_)] ×100. One hundred binding units were used as the cut-off value (19).

### Interferon-γ (IFN-γ) ELISPOT Assay

EBP-1-specific T cell responses were detected using a human IFN-γ ELISPOT Kit (MabTech, Stockholm, Sweden). Briefly, PBMCs (3×10^5^ cells/well) freshly isolated from CHB patients or HCs were transferred to an ELISPOT plate coated with antibody and blocked with 10% fetal calf serum in RPMI 1640, followed by stimulation with 5 μmol/L rhEBP-1 or negative control recombinant ovalbumin (rOVA, purity > 95%; Creative Diagnostics, NY, USA). Cells cultured in RPMI 1640 were used as negative controls. After 36 h of incubation at 37°C and washing, plates were sequentially incubated with a biotinylated-anti-human IFN-γ monoclonal antibody and streptavidin-alkaline phosphatase at room temperature. After washing, 100 μL NBT/BCIP (Pierce, Rockford, IL, USA) was added to each well and incubated at room temperature until the spots became visible. The spots were counted using a ChampSpot™ ELISPOT reader (Saizhi, Beijing, China). The number of spots per well was normalized to 1 × 10^5^ cells and averaged. The stimulation index (SI) was calculated as the mean number of spots in test wells divided by the mean number of spots in control wells. Wells with no spots were assigned a value of 1. A specific response was considered significant when the mean number of spots ≥ 10/10^5^ cells and SI ≥ 2.5 (20).

### EBP-1 Peptides Pool

The EBP-1 peptides pool (**Supplemental Table 1**), which consisted of 47 peptides (18-mer overlapping with 11 aa) covering the entire sequence of human EBP-1 protein, were custom synthesized with ≥90% purity by Chinese Peptide Company. A working stock containing 100mg/ml of each peptide was prepared in 1% dimethylsulfoxide/Rosewell Park Memorial Institute (RPMI).

### Flow Cytometry Analysis

PBMCs were stimulated with or without EBP-1 peptide pool (the final concentration for each peptide was 2 μg/mL) in the presence of 1 μg/mL anti-CD28 and anti-CD49d antibodies (BD Biosciences, San Jose, CA, USA). After 2 h of incubation at 37°C, 0.65 μL/mL GolgiStop™ (BD Biosciences) was added to inhibit cytokine secretion for an additional 4 h. Cells were then harvested, stained with PerCP-Cy5.5-conjugated anti-human CD4 (eBioscience, USA), and fixed, followed by permeabilization and staining with APC-labelled anti-human IFN-γ or PE-labelled anti-human IL-17 (eBioscience). Fluorescein-conjugated isotype antibodies were used as negative controls. Flow cytometry analysis was performed using a FlowJo software (version 7.6.3; Treestar, USA).

### Statistical Analysis

Statistical analysis was performed using SPSS 13.0 (IBM, Armonk, NY, USA). Graphs were generated using Prism 5.0 (GraphPad, San Diego, CA, USA). Differences among two groups were compared using non-parametric Mann Whitney test. Differences among multiple groups were compared using a non-parametric ANOVA (Kruskal-Wallis test) followed by Dunn’s Multiple Comparison Test. The correlation between the level of serum anti-EBP-1 autoantibody and ALT, TB, or DNA levels was calculated using a linear regression model. A *P* value < 0.05 was considered statistically significant.

## Results

### Identification of the Liver Proteins Recognized by Serum Autoantibodies From Chinese CHB Patients

**Figure 1:**
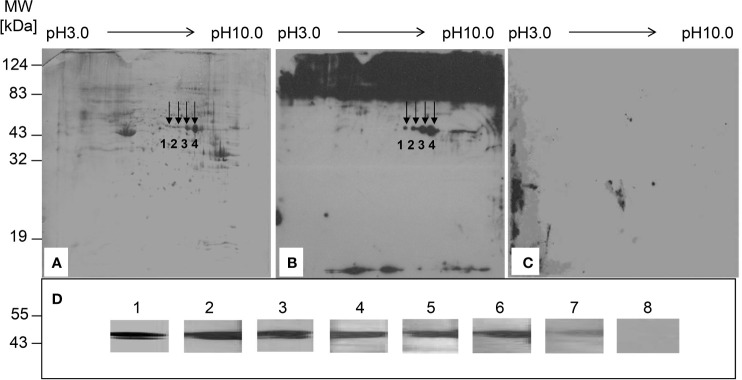
Identification of the liver proteins recognized by serum autoantibodies from patients with chronic hepatitis B (CHB). **(A)** Proteins from HepG-2 cells were separated by two-dimensional electrophoresis and visualized by Coomassie blue staining. **(B)** Proteins from HepG-2 cells were transferred to a PVDF membrane, followed by incubation with a mixture of diluted sera samples (1:100) from 20 patients with CHB. **(C)** PVDF membrane was incubated with a mixture of diluted serum samples (1:100) from 15 healthy controls (HCs). Arrows indicate the proteins recognized by serum samples from patients with CHB. **(D)** Western blot analysis was performed to detect recombinant human EBP-1 using polyclonal anti-EBP-1 antibody (1:500) (lane 1) or diluted sera (1:100) from CHB patients (lane 2–7) or HC (lane 8).

To identify the liver proteins recognized by serum autoantibodies from Chinese CHB patients, proteins from HepG-2 cells were separated by 2-DE (**Figure 1A**), transferred to a PVDF membrane, and incubated with a mixture of serum samples from CHB patients or HCs. The reactivity of patients’ sera with proteins from HepG-2 cells resulted in 4 spots (**Figure 1B**). No reactive protein spots were observed in the HC samples (**Figure 1C**). Mass spectrometry revealed that the immunoreactive proteins were serum albumin (spot 1), EBP-1 (spot 2), and alpha-enolase (spots 3 and 4) (**Supplemental Table 2**). Since albumin and alpha-enolase are established autoantigens in chronic liver diseases (21, 22), we further investigated EBP-1 in the following experiments. Western blot analysis showed that rhEBP-1 protein was detected by serum autoantibodies from CHB patients (**Figure 1D**, lane 2–7), but not by those from HCs (**Figure 1D**, lane 8), further indicating the presence of autoantibody against EBP-1 in the sera from CHB patients.

#### Hepatic EBP-1 Protein Expression is Upregulated in CHB Patients With High-Grade Hepatic Inflammation

**Figure 2:**
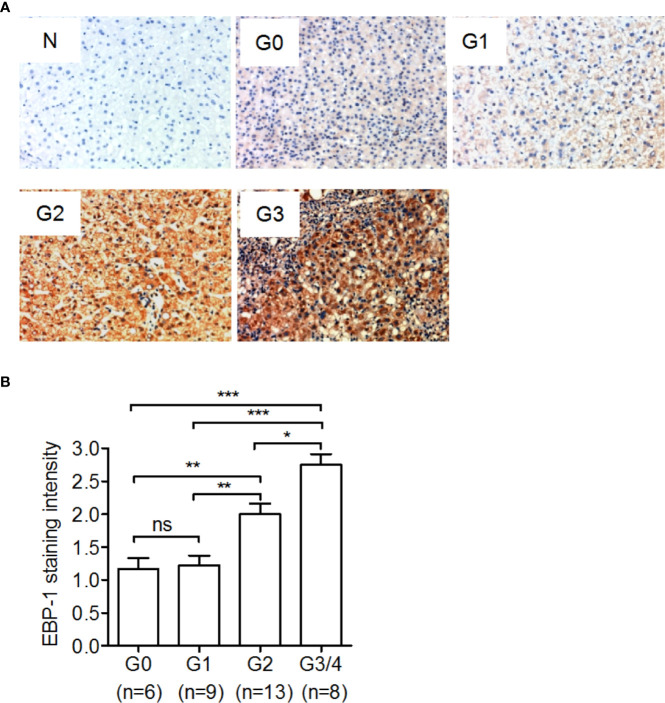
Immunohistochemical staining of ErbB-3-binding protein-1 (EBP-1) in liver tissue samples from CHB patients. A total of 36 CHB patients underwent liver biopsy, and immunohistochemical staining was performed to detect EBP-1 expression in the liver tissue samples. Hepatic inflammation was graded as G0, G1, G2, and G3-G4 using the modified histological activity index. **(A)** Representative images of immunohistochemical staining of EBP-1 in liver tissue samples with different histological grades. The intensity of EBP-1 staining in liver tissue incubated with normal rabbit IgG (1:200) was used as a negative control (N). Magnification 200 ×. **(B)** The intensity of EBP-1 was scored as follows: 0 = negative, 1 = weak, 2 = moderate, and 3 = strong. The EBP-1 staining scores were compared among CHB patients with different inflammatory grades. Significance was determined by Kruskal-Wallis test followed by Dunn’s Multiple Comparison Test; ns, non-significant; *P < 0.05; **P <0.01; ***P < 0.001.

To investigate the association of EBP-1 expression with hepatic inflammation in CHB patients, we performed IHC to detect hepatic EBP-1 expression in 36 CHB patients with different grades of hepatic inflammation. As shown in **Figures 2A, B**, despite comparable staining intensities of hepatic EBP-1 between the patients with inflammatory grades 0 and 1, the EBP-1 staining intensities were significantly increased across grades 1 to 4 in a grade-dependent manner. This finding suggests that EBP-1 protein expression might positively correlates with the severity of hepatic inflammation in CHB patients.

### The Prevalence and Levels of Serum Anti-EBP-1 IgG Are Increased in CHB Patients With Severe Diseases

**Figure 3:**
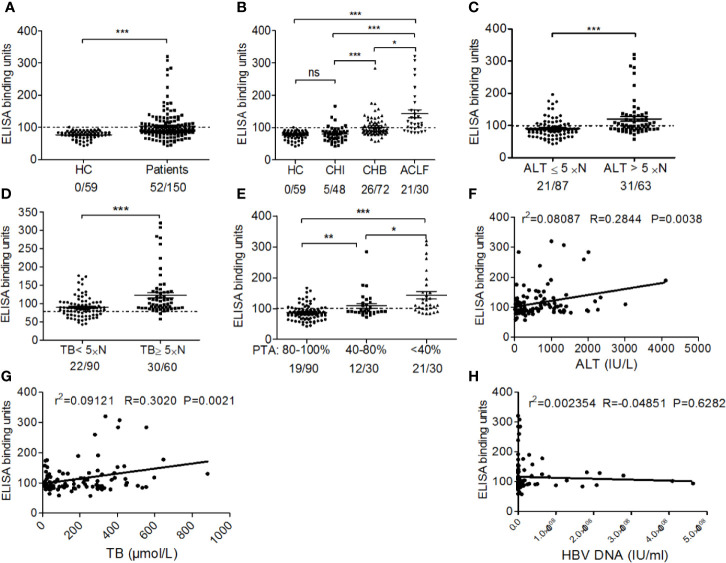
The association of prevalence and titres of anti-EBP-1 autoantibodies with clinical features of patients with CHB. **(A)** The scatter plot showing the comparison of ELISA results of serum anti-EBP-1 autoantibodies in total patients with chronic HBV infection (n=150) versus HC(n=59); Significance was determined by Mann Whitney test. **(B)** Comparison of the prevalence and titres of serum anti-EBP-1 autoantibodies in HCs (n = 59) and patients with CHI (n = 48), CHB (n = 72) or ACLF (n = 30); X/X = the number of EBP-1 autoantibody-positive patients/the total number of patients in each group. HC: healthy control; CHI: eAg +/- chronic HBV infection; CHB: eAg +/- chronic hepatitis B; ACLF: HBV-associated acute-on-chronic liver failure. Significance was determined by Kruskal-Wallis test followed by Dunn’s Multiple Comparison Test. Comparison of the prevalence and titres of serum anti-EBP-1 autoantibodies in patients with **(C)** low (≤ 5 × normal value; 21/87) and high (> 5× normal value; 31/63) ALT levels, **(D)** low (< 5 × normal value; 18/90) and high (≥ 5 × normal value; 30/60) TB levels (Mann Whitney test), **(E)** low (< 40%; 21/30), moderate (40–80%; 12/30), and high (80–100%; 19/90) PTA (Kruskal-Wallis test followed by Dunn’s Multiple Comparison Test). The dotted line at 100 binding units represents the cut-off value. The scatter plot showing the correlation between serum anti-EBP-1 autoantibody levels and **(F)** ALT, **(G)** TB, or **(H)** HBV DNA levels. ALT, alanine aminotransferase; TB, total bilirubin; PTA, prothrombin activity; ns, non-significant; *P < 0.05; **P < 0.01; ***P < 0.001.

Using ELISA, we found that anti-EBP-1 IgG were detectable in the serum samples of 34.67% (52/150) untreated patients with CHB, but in none of the HCs (0/59) (**Figure 3A**). Then, we sought to explore whether serum anti-EBP1 IgG is associated with disease severity in patients. After dividing all patients into chronic HBV infection (CHI; n = 48), chronic hepatitis B (CHB; n = 72), and HBV-associated acute-on-chronic liver failure (ACLF; n = 30) groups according to the disease severity (**Table 1**), we found that the CHI group displayed increased anti-EBP-1 prevalence (CHI: 4/48 vs HC: 0/59), but not the titres, compared with HCs; CHB and ACLF groups had significantly increased prevalence and titres of serum anti-EBP-1 IgG compared with HCs, with the ACLF group showing the highest prevalence and titres of anti-EBP-1 IgG among all groups (**Figure 3B**). Furthermore, compared with patients with low ALT levels (≤5 × normal value), patients with high ALT levels (> 5 × normal value) exhibited significantly increased prevalence and titres of serum anti-EBP-1 IgG (**Figure 3C**). Similar results were observed in patients with high TB levels compared with patients with low TB levels (**Figure 3D**). The 52 CHB patients with lower PTA had significantly increased prevalence and levels of serum anti-EBP-1 IgG compared with those with higher PTA (**Figure 3E**). In addition, both serum ALT and TB levels were positively correlated with anti-EBP-1 autoantibodies (**Figures 3F, G**), but there was no correlation between HBV DNA levels and serum anti-EBP-1 autoantibody levels (**Figure 3H**). These data collectively suggest that the prevalence and levels of serum anti-EBP-1 IgG are positively associated with disease severity in CHB patients.

### EBP-1 Antigens Induce IFN-γ Release From PBMCs of CHB Patients

**Figure 4:**
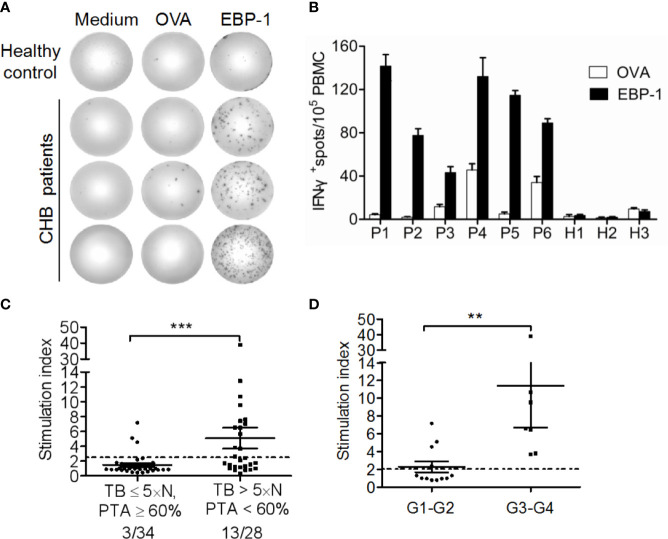
IFN-γ ELISPOT assay for detection of EBP-1-specific T cell-responses. **(A)** Representative ELISPOT wells showing IFN-γ-production by PBMCs from one HC and three CHB patients stimulated with recombinant EBP-1, OVA or medium. **(B)** The representative number of IFN-γ^+^ spots/1 × 10^5^ PBMCs from 6 CHB patients and 3 HCs. Data are expressed as the mean ± standard deviation (SD). **(C)** The stimulation indexes (SI) and the percentages of positive responders were compared between patients with severe (TB > 5×N, PTA < 60%) and weak or moderate (TB ≤ 5×N, PTA ≥ 60%) liver injury. **(D)** The SIs were compared between patients with histological inflammation grade 1–2 (G1–G2) and G3–G4. Data are expressed as the mean ± SD. Significance was determined by Mann Whitney test, **P < 0.01; ***P < 0.001.

To explore whether EBP-1 could induce autoreactive T cell response in CHB patients, we performed ELISPOT assays to detect IFN-γ production in PBMCs from CHB patients or HCs in response to EBP-1 or control antigen rOVA stimulation *in vitro*. We used recombinant human EBP-1 proteins (purity > 95%) to stimulate PBMCs from CHB patients and HCs regardless of the human leukocyte antigen (HLA) types. We found that only PBMCs from CHB patients produced considerable amount of IFN-γ in response to EBP-1 peptide stimulation (**Figures 4A, B**). Of note, the percentage of patients with severe liver injury (TB > 5 × normal value, PTA < 60%) responded to rhEBP-1 proteins was significantly greater than the percentage of patients with weak or moderate liver injury (TB ≤ 5 × normal value, PTA ≥ 60%; 46.43% vs. 8.82%, *P* < 0.05; **Figure 4C**). In addition, the SI that indicates the amount of IFN-γ production was significantly increased in the patients with severe diseases compared with that in the patients with weak or moderate diseases (**Figure 4C**). Similarly, patients with higher HAI scores had remarkably greater SIs than those with lower HAI scores (**Figure 4D**). Although not all PBMCs from anti-EBP-1 IgG-positive patients had detectable IFN-γ production in response to rhEBP-1 stimulation, these data still suggest that EBP-1-specific T-cell IFN-γ response exists in the peripheral blood of CHB patients and might positively correlates with the severity of liver disease.

### EBP-1 Peptides Induce IFN-γ and IL-17 Expression in CD4^+^ T Cells From CHB Patients

**Figure 5:**
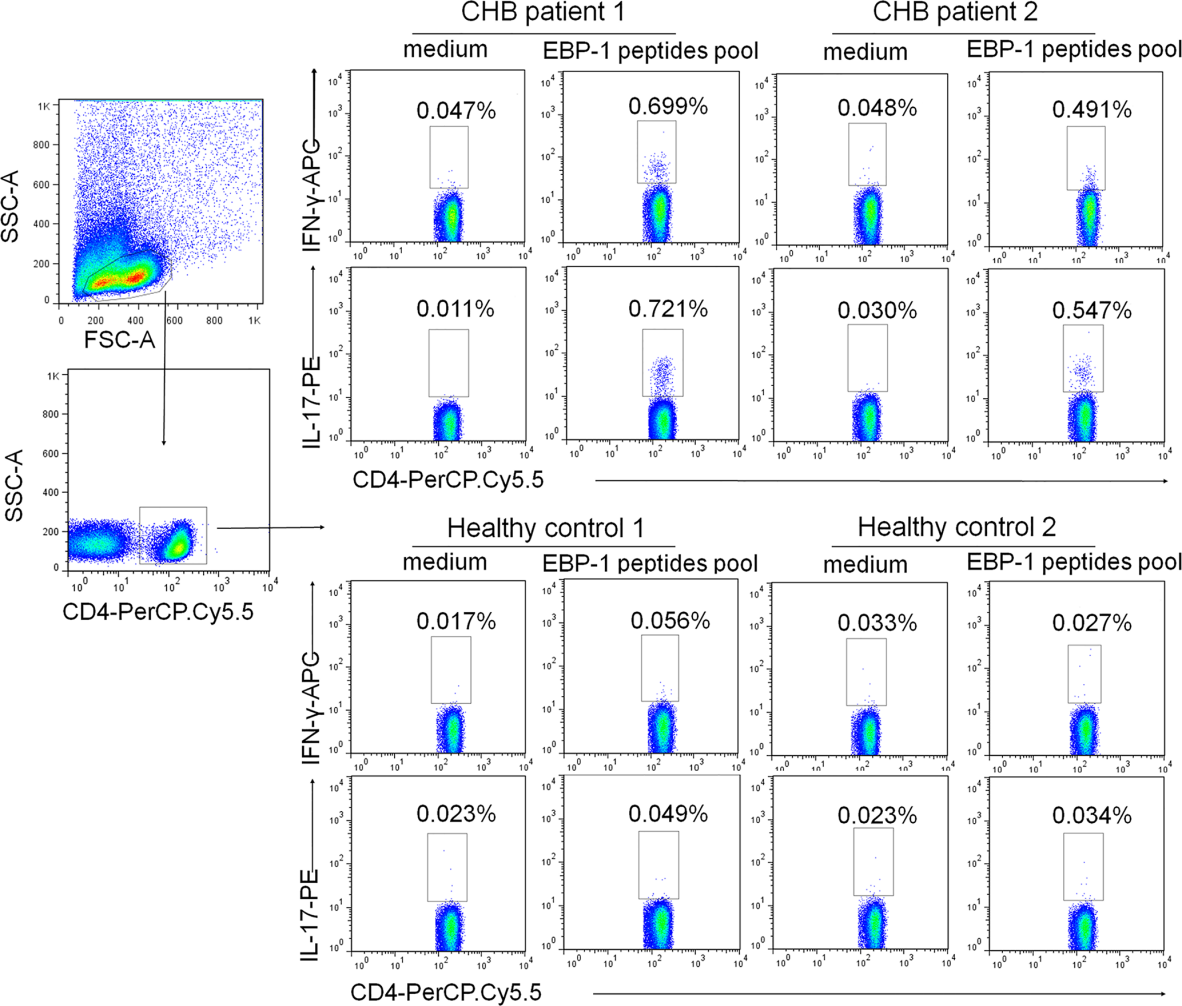
EBP-1 peptides induced IFN-γ and IL-17 expression in CD4^+^ T cells from CHB patients. PBMCs from 2 CHB patients and 2 HCs were stimulated with or without a synthetic EBP-1 peptides pool (18-mers overlapping by 11 amino acids, approximately 2 μg/mL for each peptide) in the presence of anti-CD28 and anti-CD49d antibodies. Representative flow cytometric dot plots show that the gated CD4^+^ cells in CHB patients express significantly increased IFN-γ and IL-17 upon EBP-1 peptides stimulation compared with those in HCs. The number in each quadrant indicates the percentage of CD4^+^ cells expressing IFN-γ or IL-17. SSC-A, side scatter area; FSC-A, forward scatter area.

To rule out T cell response to impurities such as bacterial or yeast proteins that are present in recombinant EBP-1 proteins, and to further evaluate EBP-1-induced autoreactive T cell responses in the peripheral circulation of CHB patients, we performed flow cytometry analysis to determine IFN-γ and IL-17 expression in CD4^+^ cells in response to a synthetic EBP-1 peptides pool, regardless of the human leukocyte antigen (HLA) types. As shown in **Figure 5**, the gated CD4^+^ cells from CHB patients expressed significantly increased levels of IFN-γ and IL-17 upon EBP-1 peptide stimulation, compared with those from HCs. These data indicate that EBP-1 also serves as an autoantigen to induce proinflammatory response in autoreactive CD4^+^ T cells from CHB patients.

## Discussion

CHB can be accompanied by autoimmunity triggered by viral infection; however, whether autoimmunity contributes to CHB pathogenesis remains largely unknown. In this study, we demonstrated for the first time that an autoantibody against EBP-1 was present in the sera of untreated Chinese CHB patients. Autoreactive inflammatory T cell responses against EBP-1 were also present in CHB patients, as evidenced by the whole EBP-1 antigen-induced significant increases in IFN-γ production by PBMCs and the EBP-1 peptides pool-stimulated IFN-γ and IL-17 expression in CD4^+^ T cells from CHB patients, compared with those from HCs. In addition, the prevalence and levels of circulating autoantibody against EBP-1 as well as EBP-1 autoreactive T cell response were positively associated with the disease severity in CHB patients. These findings suggest that EBP-1-induced autoimmune response possibly contributes to the progression of liver damage in CHB.

The primary human hepatocytes (PHH) are considered as the gold standard for identification of autoantigens derived from hepatocytes. However, it is rare to obtain normal human liver tissue through surgery and PHH may be dedifferentiated under long-term culture conditions *in vitro*, which limit the routine application of PHH. HepG2 cell line is a well-differentiated human liver carcinoma cell line, which reflects some aspects of the adult human liver and has been extensively used for identification of hepatic proteome due to its primary hepatocyte characteristics (23, 24). Here, our 2-DE-based serologic proteomic analysis identified albumin, alpha-enolase, and EBP-1 as potential autoantigens from HepG2 cells recognized by CHB patient sera. Consistent with our findings, the serum levels of anti-albumin autoantibody are elevated in CHB patients, but not associated with liver injury (21). Alpha-enolase has been identified as a major autoreactive target in patients with AIH (22) and inflammatory bowel disease (25). In this study, we focused on EBP-1 because it has not been reported elsewhere as an autoantigen in CHB. Previous studies have reported hepatocyte-derived autoantigens, including asialoglycoprotein receptor (26), hepalaminin, HSP 60 and 70 (27), in AIH, CHB, and CHC. But our 2-DE study did not confirm the presence of these autoantigens in CHB patients. This discrepancy may result from the methodological differences and HLA variations in CHB population from different areas. In addition, there are still some differences between HepG2 cells and PHH. For example, HepG2 is not suitable for studying autoreactivity against some targets whose expression are weakened in this cell line [e.g. cytochrome P450 2D6, the target of LKM1 autoantibody identified in AIH patients (28)].

EBP-1 belongs to the family of proliferation-associated 2G4 proteins and is extensively expressed in human tissues, including lung, breast, kidney, and liver, playing important regulatory roles in eukaryotic cell growth, proliferation, differentiation, and survival (29, 30). In this study, we found that CHB patients with high-grade hepatic inflammation had significantly increased EBP-1 protein levels compared with those with low-grade hepatic inflammation, suggesting that of EBP-1 upregulation might be associated with liver inflammation in CHB patients. It has been reported that HBV X protein upregulates EBP-1 expression in different cell types through E2F transcription factor 1 (31, 32), suggesting that HBV infection enhances EBP-1 expression in hepatocytes. In addition, EBP-1 is overexpressed in colorectal cancer tissue compared with that in adjacent normal tissue, resulting in immunogenic response in colorectal cancer patients (33). Thus we speculate that overexpression of EBP-1 in hepatocytes might lead to epitope spreading and a break of immune tolerance to EBP-1 in CHB.

Since hepatic EBP-1 expression was significantly increased in an inflammatory grade-dependent manner in CHB, a large amount of EBP-1 proteins might be presented by major histocompatibility complex (MHC) molecules or released from dead hepatocytes, leading to autoantibody production and autoreactive T cell responses. This process may play an important role in the initiation and development of hepatic damage in CHB. Indeed, our results showed that CHB patients with severe liver diseases had higher prevalence and serum levels of anti-EBP-1 autoantibody compared with those with mild diseases, suggesting that anti-EBP-1 autoantibody level is positively associated with liver damage in CHB. However, anti-EBP-1 autoantibodies were also detected in the serum of some patients with other liver diseases (such as AIH and CHC) (**Supplementary Figure 1**). Therefore, we speculate that EBP-1 may be a universal autoantigen associated with chronic liver inflammatory diseases, rather than a specific autoantigen of CHB. Further studies are needed to address the role of autoreactivity to EBP-1 in these liver diseases.

Our results showed that EBP-1 proteins stimulated IFN-γ production by PBMCs from CHB patients but not HCs. CHB patients with severe diseases exhibited significantly increased response rate to EBP-1 stimulation and elevated amount of IFN-γ production (SI) compared with patients with mild diseases. In addition, CD4^+^ T cells from CHB patients, but not HCs, expressed proinflammatory cytokines IFN-γ and IL-17 upon stimulation with the EBP-1 peptides pool. These data suggest that EBP-1 is a potential trigger of autoreactive T cells in CHB patients and EBP-1-induced autoimmune response is positively associated with the severity of diseases.

Excessive and non-HBV-specific Th1 and Th17 responses are involved in liver inflammation and hepatocellular damage in CHB (34). A recent study also has demonstrated that T cell recruitment to a site of inflammation is strictly dependent on local display of cognate ligands (35). Since non-HBV-specific T cells are largely found in the liver tissue of CHB patients with massive hepatic necroinflammation, we hypothesize that the bystander T cells infiltrating into the inflamed liver tissue may recognize liver autoantigens, such as EBP-1, and contribute to liver damage in CHB. However, our study did not demonstrate the infiltration of EBP-1-autoreactive T cells into the inflamed liver tissue due to a lack of fresh liver biopsy tissues from the CHB patients. In addition, we did not provide direct evidence that anti-EBP-1 autoantibody and T-cell responses are responsible for CHB liver injury, which will be addressed in future study.

In conclusion, using proteomic techniques, we identified that EBP-1 is a nonspecific autoantigen associated with chronic HBV infection. Serum autoantibodies and autoreactive T cells against EBP-1 were present in patients with chronic HBV infection, and their levels were related to the clinical severity of liver disease, indicating their possible pathophysiologial role in the development of progressive liver failure. Further prospective studies are needed to confirm that anti-EBP-1 autoimmunity is implicated in disease process. If that’s true, targeting EBP-1-mediated autoimmune response might represent as a potential immunotherapeutic strategy for CHB treatment.

## Data Availability Statement 

The original contributions presented in the study are included in the article/**Supplementary Material**. Further inquiries can be directed to the corresponding authors.

## Ethics Statement

The studies involving human participants were reviewed and approved by the Ethics Committee of Southwest Hospital (Chongqing, China). The patients/participants provided their written informed consent to participate in this study.

## Author Contributions

LJ, WN, and QZ performed sample collection, main experiments and data analysis, and draft the manuscript. GM completed immunohistochemical staining and pathological analysis. XC and MZ completed ELISPOT assay and flow cytometry analysis. QM completed patient data analysis. GD participated in guiding the project design and supervising the experimental process. LW carried out the project design, guided the research process, supervised and managed it, completed the manuscript and provided research funds. All authors contributed to the article and approved the submitted version.

## Funding

This work was supported by grants from the National Natural Science Foundation of China [grant numbers 81930061 & 82071825], and the Chongqing Natural Science Foundation [CSTC2019jcyj-zdxmX0004].

## Conflict of Interest

The authors declare that the research was conducted in the absence of any commercial or financial relationships that could be construed as a potential conflict of interest.

## Abbreviations

CHB, chronic hepatitis B; ELISA, enzyme-linked immunosorbent assay; EBP-1, ErbB-3-binding protein-1; HBV, Hepatitis B virus; ALT, alanine aminotransferase; TB, total bilirubin; PTA, prothrombin time activity; AIH, autoimmune hepatitis; HCs, healthy controls; PBMCs, peripheral blood mononuclear cells; IHC, immunohistochemical; HAI, histological activity index; DMEM, Dulbecco’s Modified Eagle’s Medium; FBS, fetal bovine serum; PBS, phosphate-buffered saline; PVDF, polyvinylidene difluoride; HRP, horseradish peroxidase; rhEBP-1, recombinant human ErbB-3-binding protein-1; SI, stimulation index; MHC, major histocompatibility complex.
