# Supplementary material for: Identification of an Autoantibody Against ErbB-3-Binding Protein-1 in the Sera of Patients With Chronic Hepatitis B Virus Infection
Source: Front Immunol. 2021 May 25;12:640335. doi: 10.3389/fimmu.2021.640335 (PMC8185336; doi:10.3389/fimmu.2021.640335)
Supplement: Supplementary file 1 [file DataSheet_1.docx]

**Supplemental data**

**Supplemental Table 1.** The sequences of 47 synthetic human EBP-1 peptides (18-mer overlapping with 11 aa)

| Synthetic peptide | Sequence |
| --- | --- |
| EBP-1_1-18_ | mimeetgkifkkekemkk |
| EBP-1_8-25_ | kifkkekemkkgiafpts |
| EBP-1_15-32_ | emkkgiafptsisvnncv |
| EBP-1_22-39_ | fptsisvnncvchfspl**k** |
| EBP-1_29-46_ | nncvchfsplksdqdyil |
| EBP-1_36-53_ | splksdqdyilkegdlvk |
| EBP-1_43-60_ | dyilkegdlvkidlgvhv |
| EBP-1_50-67_ | dlvkidlgvhvdgfianv |
| EBP-1_57-74_ | gvhvdgfianvahtfvvd |
| EBP-1_64-81_ | ianvahtfvvdvaqgtqv |
| EBP-1_71-88_ | fvvdvaqgtqvtgrkadv |
| EBP-1_78-95_ | gtqvtgrkadvikaahlc |
| EBP-1_85-102_ | kadvikaahlcaeaalrl |
| EBP-1_92-109_ | ahlcaeaalrlvkpgnqn |
| EBP-1_99-116_ | alrlvkpgnqntqvteaw |
| EBP-1_106-123_ | gnqntqvteawnkvahsf |
| EBP-1_113-130_ | teawnkvahsfnctpieg |
| EBP-1_120-137_ | ahsfnctpiegmlshqlk |
| EBP-1_127-144_ | piegmlshqlkqhvidge |
| EBP-1_134-151_ | hqlkqhvidgektiiqnp |
| EBP-1_141-158_ | idgektiiqnptdqqkkd |
| EBP-1_148-165_ | iqnptdqqkkdhekaefe |
| EBP-1_155-172_ | qkkdhekaefevhevyav |
| EBP-1_162-179_ | aefevhevyavdvlvssg |
| EBP-1_169-186_ | vyavdvlvssgegkakda |
| EBP-1_176-193_ | vssgegkakdagqrttiy |
| EBP-1_183-200_ | akdagqrttiykrdpskq |
| EBP-1_190-207_ | ttiykrdpskqyglkmkt |
| EBP-1_197-214_ | pskqyglkmktsraffse |
| EBP-1_204-221_ | kmktsraffseverrfda |
| EBP-1_211-228_ | ffseverrfdampftlra |
| EBP-1_218-235_ | rfdampftlrafedekka |
| EBP-1_225-242_ | tlrafedekkarmgvvec |
| EBP-1_232-249_ | ekkarmgvvecakhellq |
| EBP-1_239-256_ | vvecakhellqpfnvlye |
| EBP-1_246-263_ | ellqpfnvlyekegefva |
| EBP-1_253-270_ | vlyekegefvaqfkftvl |
| EBP-1_260-277_ | efvaqfkftvllmpngpm |
| EBP-1_267-284_ | ftvllmpngpmritsgpf |
| EBP-1_274-291_ | ngpmritsgpfepdlyks |
| EBP-1_281-298_ | sgpfepdlyksemevqda |
| EBP-1_288-305_ | lyksemevqdaelkallq |
| EBP-1_295-312_ | vqdaelkallqssasrkt |
| EBP-1_302-319_ | allqssasrktqkkkkk |
| EBP-1_309-326_ | srktqkkkkkkasktaen |
| EBP-1_316-333_ | kkkkasktaenatsgetl |
| EBP-1_323-340_ | taenatsgetleeneagd |

**Supplemental Table 2.** Summary of proteins identified using two-dimensional electrophoresis/MALDI-TOF (Spectra were analyzed using the Mascot Search engine that queried the Swiss-Prot database).

| Spot | Protein | UniProt/ Swiss-Prot identifier | % Protein covered | Molecular mass (kDa) | | PI | |
| --- | --- | --- | --- | --- | --- | --- | --- |
|  |  |  |  | Theoretical | Observed | Theoretical | Observed |
| 1 | Serum albumin | P02768-1 | 26.11 | 69.4 | 43-60 | 5.92 | 5.8-6.0 |
| 2 | EBP-1 | Q9UQ80 | 47.21 | 48 | 43-60 | 6.13 | 6.0-6.2 |
| 3 | Alpha-enolase | P06733-1 | 64.52 | 47.2 | 43-60 | 7.01 | 7 |
| 4 | Alpha-enolase | P06733-1 | 74.19 | 47.2 | 43-60 | 7.01 | 8 |

**Supplemental Figure. 1**


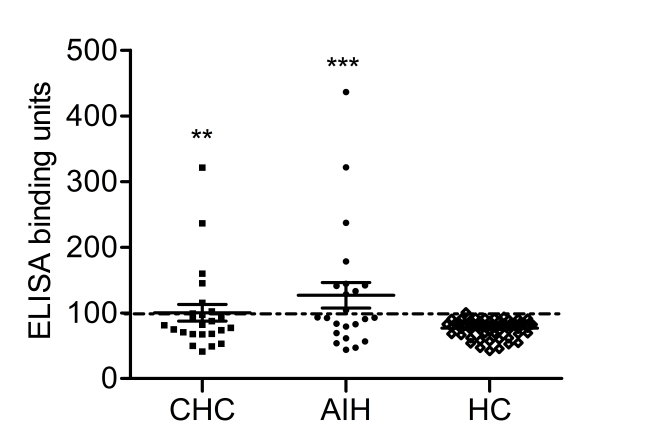


**Supplemental Figure. 1.** The prevalence and titres of anti-EBP-1 autoantibodies in patients with CHC, AIH and HCs.

Purified recombinant EBP-1 was used as antigen in ELISAs. Antibody titres were calculated as binding units according to the formula shown in Methods. One hundred binding units was used as the cut-off (see text). Statistically significant differences between ELISA binding units between each patient group and HCs are indicated as follow: **p <0.01; ***p <0.001.
